# To the Editors of the Medical and Physical Journal

**Published:** 1808-11

**Authors:** R. Hall

**Affiliations:** Clifford's Inn


### *To the Editors of the Medical and Physical Journal.*

Gentlemen,

In the nineteenth volume of the Medical and Physical Journal, page 119, an obstinate case of Frambœsia, or Yaws, is mentioned, in which the disease, though of long standing, is said to have been cured by the use of a remedy, which the author considers as new and unknown to medical practitioners. The remedy however therein recommended has been long employed in this complaint in several of the French West India Islands, as I have been informed by a medical gentleman, who resided for many years in that part of the world. What moreover tends to confirm the above account is the testimony of Pere Labat, who, when speaking on this subject, in his Histoire de la Louisiane, mentions the following formula, which the reader will observe, differs not essentially from that to which the cure in the above case is principally attributed.

"Take iron rust reduced to powder, and passed through a fine sieve; moisten it with citron juice, so that it may have the consistence of ointment; spread it on a linen rag, rubbed over with wheel grease, or fresh lard, and apply it night and morning."

Without calling in question the efficacy of the carbonate and oxides of iron in phagedenic and ill-conditioned ulcers,* we may be permitted to observe, that the total want of information respecting the regimen employed in the case of this unfortunate patient, greatly detracts from the value of Dr. Humphrey's communication.

It is well known that nothing proves more conducive to the cure of this loathsome disorder, than a nutritious regimen, attention to cleanliness, pure air, and sea bathing; yet in the case under consideration, excepting a decoction of some roots, exhibited occasionally to the patient by his countrymen, and which the author supposes to have operated as a purgative, we are not informed whether any internal remedies were employed, or any measures pursued to improve the general health, or invigorate the constitution,

* A case lately fell under my own observation, and which will probably appear in a future number of the Medical Journal, of a phagedenic ulcer of the tongue, which readily yielded to the carbonate of iron, after resisting various other remedies.

tion, though every practitioner, acquainted with the connection between local and general disease, knows that unless attention be given to correct the cachectic state, or bad habit of the patient, labouring under ill-conditioned ulcers, all local means will for the most part prove ineffectual to promote their cicatrization

I am, &c.

R. HALL.

*Clifford's Inn, 1st October, 1808.*

---

